# Relationship Between Macrophage Arc and Plaque Vulnerability in Patients With Coronary Artery Disease

**DOI:** 10.1016/j.jacadv.2026.103167

**Published:** 2026-08-19

**Authors:** Zelin Ou, Xiaolong Qu, Bin Cui, Li Xie, Ke Wang, Han Xiao, Yili Ma, Maoyu Zhao, Youzhu Qiu, Yao Zhang, Dehui Qian, Zhihui Zhang, Bin Liu, Xiaohui Zhao

**Affiliations:** a Department of Cardiology, Xinqiao Hospital, Army Medical University, Chongqing, China; b Department of Cardiovascular Medicine, Southwest Hospital, Army Medical University, Key Laboratory of Geriatric Cardiovascular and Cerebrovascular Disease, Ministry of Education of China, Chongqing, China; c Department of Statistics, Army Medical University, Chongqing, China; d Department of Cardiology, Secondary Hospital, Jilin University, Jilin Province, China; e Department of Geriatrics, Xinqiao Hospital, Army Medical University, Chongqing, China

**Keywords:** acute myocardial infarction (AMI), macrophage arc, optical coherence tomography (OCT), vulnerable plaque

## Abstract

**Background:**

Macrophages are key drivers of coronary artery plaque development and can be detected via optical coherence tomography as circumferential regions. However, the association between macrophage arc and plaque vulnerability remains unclear.

**Objectives:**

The objective of the study was to evaluate the correlation between macrophage arc and vulnerable plaques.

**Methods:**

This multicenter, retrospective study enrolled consecutive patients with coronary artery disease who underwent optical coherence tomography (January 2017–April 2023). Macrophage arc was evaluated using maximum arc, mean arc, and mean arc score (MAS) in the target vessel. The mean arc and MAS were calculated as the total detectable macrophage arc or score of each frame, divided by imaged vessel segment length (mm). Furthermore, the association between macrophage arc, plaque vulnerability, and acute myocardial infarction (AMI) at admission was investigated.

**Results:**

Overall, 1,129 patients (1,278 vessels; 1,525 vulnerable plaques) were enrolled. The macrophage arc was significantly increased in cases of plaque rupture (PR), thin-cap fibroatheroma (TCFA), and AMI cases (*P* < 0.0001). Both the mean arc and MAS were markedly higher in regions adjacent to PR or TCFA compared with the entire segment (*P* < 0.0001). Multivariate analyses demonstrated that maximum arc, mean arc, and MAS were correlated with PR, TCFA, and AMI. Receiver operating characteristic analysis identified optimal cutoff values—maximum arc ≥156.5°, mean arc ≥78.89°/mm, and MAS ≥2.34—each significantly associated with PR, TCFA, and AMI, respectively (all *P* < 0.001).

**Conclusions:**

Macrophage arcs are associated with plaque vulnerability and AMI. Further external validation and prospective studies are needed to confirm these observations.

Early identification of vulnerable plaques, which are coronary artery lesions characterized by thin-cap fibroatheroma (TCFA) or plaque rupture (PR), is clinically important and has attracted considerable attention in cardiovascular research.^1^^,^^2^ Proinflammatory macrophages play a critical role in plaque destabilization by secreting matrix-degrading enzymes and contributing to structural weakening.^3^ Consequently, imaging macrophages may aid in the early identification of patients with vulnerable plaques who are at an increased risk of acute myocardial infarction (AMI).^4^, ^5^, ^6^, ^7^, ^8^

Optical coherence tomography (OCT) is a powerful imaging modality capable of identifying macrophages in biological tissues. In atherosclerotic plaques, macrophages appear as distinct or confluent punctate regions with high-intensity signals, markedly stronger than background speckle noise.^9^ To quantify macrophage presence, Tearney et al^10^ introduced the normalized SD (NSD), which has demonstrated utility in evaluating macrophage density.^11^ Studies have shown that culprit plaques exhibit higher macrophage density, as measured by NSD, compared with nonculprit plaques,^12^ and that patients with ST-segment elevation myocardial infarction have greater morphological density than those with stable angina.^13^ However, dedicated software for NSD analysis has not been available in commercial OCT systems for over 2 decades, and its clinical application has largely been restricted to preselected regions of interest, mainly within the fibrous cap of fibroatheromas, which may not fully represent the overall macrophage distribution.^10^ Furthermore, NSD reliability is limited by multiple factors: 1) other plaque tissue components can generate high NSD values, interfering with the accurate assessment of macrophage-related signals; and 2) noise-reduction processes in OCT images may inadvertently remove important macrophage-related information. These limitations underscore the need for improved methods and technologies to accurately quantify macrophages using OCT in clinical practice.^11^

During OCT imaging, macrophages tend to aggregate and form clusters that appear as highly reflective tissue bands, standing out prominently in the OCT images. A post hoc analysis from the CLIMA study revealed that quantitative assessment of the circumferential arc of macrophage extension can be performed with high reproducibility.^14^ This approach may provide a valuable tool for the prompt detection of macrophage infiltration, potentially enhancing the evaluation of atherosclerotic plaque vulnerability and guiding clinical decision-making.^15^^,^^16^ However, the precise relationship between macrophage arc and plaque vulnerability remains uncertain. The primary objective of this study was to evaluate the correlation between macrophage arc and features of vulnerable plaques, including TCFA, PR, intra-plaque bleeding, cholesterol crystals, erosion, calcified nodules, plaque disruption, and microvessels. In addition, the study aimed to assess the relationship between macrophage arc and AMI at admission, defined as positive troponin results within 7 days of admission.

## Methods

### Study design

This retrospective, multicenter registry study was conducted between January 2017 and April 2023 and included patients with coronary artery disease who underwent coronary angiography and OCT at Xinqiao Hospital and Southwest Hospital of the Army Medical University in Chongqing, China. Patients with poor OCT image quality or with an effective statistical vessel length of <20 mm were excluded.

### Data collection and processing

Data collection was conducted in strict accordance with institutional regulations using the hospital’s electronic medical record system. Rigorously defined protocols were followed during OCT data analysis to ensure both accuracy and reliability. If prepercutaneous coronary intervention OCT data were available, they were preferentially selected for statistical analysis; otherwise, data from nonstented segments were used. For long vessels that underwent double OCT examinations, continuous statistical processing was applied. The study was approved by the local ethics committee and registered in the Chinese Clinical Trial Registry (ChicCTR2400091532). The authors assume full responsibility for the study’s design, execution, conduct, and final contents. A STROBE (Strengthening the Reporting of Observational Studies in Epidemiology) checklist for retrospective observational studies is completed (Supplemental Appendix).

### Imaging acquisition

#### OCT data and analysis

Coronary angiographies were performed using standard techniques, whereas OCT images were acquired with the ILUMIEN OPTIS system or OPTIS system (Abbott) using a nonocclusive approach. Full-vessel plaque feature annotation was carried out by 2 independent investigators (O.Z.L and Q.X.L.) who did not participate in macrophage arc measurements. OCT image analysis was performed by 2 experienced investigators (X.L. and X.H.) blinded to the clinical presentation and plaque features; discrepancies were adjudicated by a third investigator (W.H.). Only OCT pull backs that allowed accurate lumen measurement (requiring a continuous arc of at least 270° around the lumen center) and qualitative assessment of superficial plaque components were deemed eligible. Specifically, plaques were included if surrounded by at least of 5 mm of healthy vessel. Frames with side branch openings, severe motion artifacts, signal dropout, or stent implantation were excluded from the adjacent region. The valid length of the adjacent segment was recalculated after excluding invalid frames, and both mean arc and mean arc score (MAS) were normalized by the valid segment length (mm) to ensure accuracy comparison with the entire vessel segment.

#### OCT parameter definition

Definitions and cutoff values for OCT parameters were based on established consensus documents and prior OCT research findings,^17^ with particular emphasis on markers of vulnerable plaques. TCFA was defined as a minimum fibrous cap thickness of <65 μm and lipid core arc extension of >180°.^18^ Microchannels were identified as clear, signal-poor cavities typically visible across multiple consecutive frames. Plaque disruption was characterized by deeper tissue with a clear layered structure. Erosions was defined as the presence of thrombus or an irregular luminal surface without cap rupture, evaluated across multiple adjacent frames. Calcified nodules were defined as nodular calcific accumulations at sites of disrupted fibrous caps on calcified plaques. Cholesterol crystals appeared as thin, linear, high-intensity regions typically associated with fibrous caps or necrotic cores. Intraplaque hemorrhage was defined as signal-poor OCT regions that required differentiation from lipid necrotic pools.^17^^,^^19^

PR was identified by consensus reading as the frame showing the most evident fibrous cap disruption and rupture ostium. The proximal and distal edges of the PR lesion were defined as the first and last frames demonstrating the rupture ostium, respectively, with the adjacent region defined as the continuous OCT frames within 5-mm proximal and 5-mm distal to these edges. TCFA was identified by consensus reading as the frame with the thinnest fibrous cap (<65 μm) and a lipid arc >180°. The proximal and distal boundaries of the TCFA lesion were defined as the first and last frames with a lipid arc exceeding 180°, respectively, and the adjacent region was defined as the continuous OCT frames within 5 mm proximal and distal to these boundaries. For all secondary plaque features (cholesterol crystals, intraplaque hemorrhage, and microchannels), the adjacent region was uniformly defined as 5 mm proximal and distal to the lesion edge, consistent with the definition for PR and TCFA.

**Figure 1:**
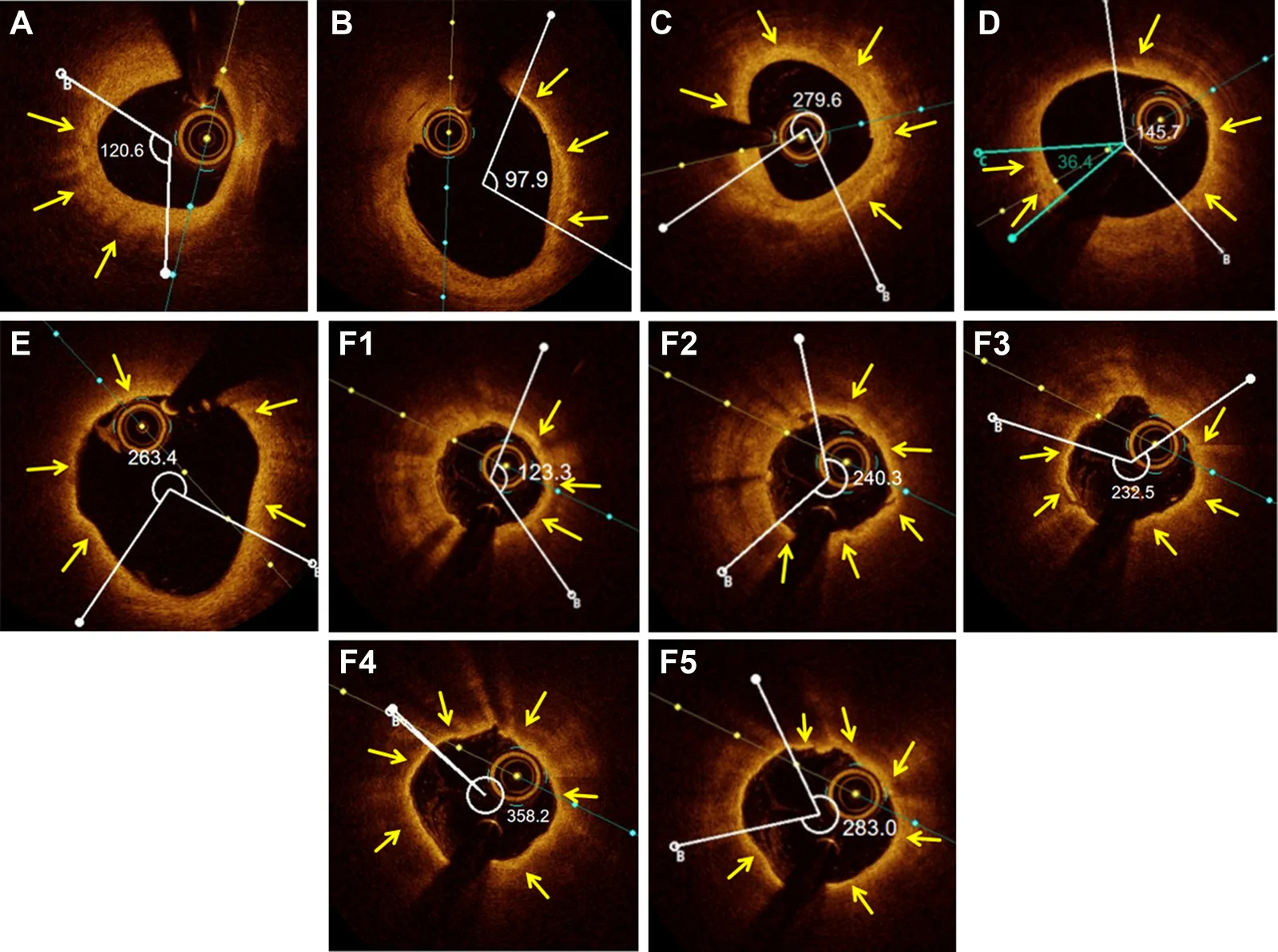
Representative Picture of Optical Coherence Tomography-Derived Macrophage Arc (A) A 120.6° raster macrophage arc (grade 3); (B) a continuous 97.9° macrophage arc (grade 3); (C) a large 279.6° macrophage arc (grade 4); (D) 2 separated macrophage clusters with a total angle of 182.1°(grade 2); (E) TCFA (lipid arc 263.4°, fiber cap thickness <0.065 mm, without macrophages infiltration); (f1-f5) sequential frame-by-frame dynamics of macrophages adjacent to a TCFA, demonstrating gradual expansion of the macrophage arc and morphological transition from dot-like to linear structures. In f1 and f2, typical macrophages are identified, with intense background speckle noise casting striated shadows. In f3, sharp transverse shadows are projected in the 1 to 10o'clock direction, and rapid frame changes suggest macrophage presence. In f4, a TCFA is formed with a macrophage arc of 358.2°. In f5, the macrophage arc decreases, and the morphology reverts to a dot-like pattern.

Macrophages were identified as signal-rich, discrete, or confluent punctate areas with intensity exceeding background speckle noise.^17^ The macrophage arc was measured using the vascular luminal center as the reference point.^14^ Frame-by-frame quantitative measurements of macrophage arc were performed across the vessels, and all values were recorded. The maximum macrophage arc was defined as the highest macrophage arc value measured across consecutive frames within the target vessel segment.^20^ A standardized standard operating procedure is provided in the Supplemental Appendix. The mean arc was computed as the sum of detectable macrophage arcs across all frames divided by the length of the imaged vessel segment (mm). The macrophage arc score was assessed using a 0 to 4 grading scale, ranging from no detectable macrophages to extensive infiltration, as described in previous research.^21^ The cumulative grade was then divided by the length of the imaged vessel segment (mm) to derive the MAS (Figure 1).

### Statistical analysis

Variables were expressed as mean ± SD for normally distributed data, median (IQR: 25th–75th percentiles) for non-normally distributed data, and categorical variables as proportions. Bivariate analyses were conducted using the Student’s *t*-test, paired samples *t*-test, Mann-Whitney *U* test, chi-square test, or Fisher exact test, as appropriate. Consistency was assessed using the kappa coefficient, and Bonferroni correction was applied to adjust the significance level (α), calculated as α = 0.05/number of comparisons to control type I error. Variables with corrected *P* < 0.05 were considered marginally significant and, owing to their clinical relevance, were prioritized for inclusion in subsequent multivariate analyses. Core covariates were predefined based on clinical relevance and previous literature, variables with *P* < 0.05 in univariate analyses were additionally incorporated to adjust for residual confounding, rather than being selected solely based on statistical significance. Each outcome predefined different analysis, plaque-level outcomes (PR and TCFA) were analyzed at the vessel level and the clinical outcome (AMI) was analyzed at the patient level. For patients with multiple vessels analyzed, the overall mean macrophage arc normalized to total vessel length was calculated, yielding a single value that reflects systemic coronary inflammatory burden. Receiver operating characteristic curves derived from raw parameters were used to evaluate the predictive performance of OCT parameters for plaque risk, with optimal cutoff values determined by maximum Youden index (J). Bivariate correlations between all enrolled study variables, high-risk plaque features, and clinical outcomes were analyzed. Variance inflation factor was calculated for all covariates in each revised model to avoid multicollinearity. Variables exhibiting marginally significant associations (*P* < 0.05) were entered into logistic hierarchical regression models to identify associated factors, and estimate adjusted ORs alongside their corresponding 95% CIs. Statistical significance was defined as a 2-tailed *P* value <0.05. Analyses were performed using SPSS software (version 24.0; IBM Corporation). The overall missingness rate for clinical and OCT variables was <3.2%. As this was a retrospective study, complete case analysis was conducted for primary and secondary analyses; missing data were determined to be completely at random and excluded.

## Results

### Demographic and procedural data

Between January 2017 and April 2023, coronary OCT was performed in 1,307 patients and 1,436 vessels across 2 independent centers. After applying exclusion criteria, 1,129 patients with 1,278 blood vessels and 1,525 vulnerable plaques were included in the analysis (PR, n = 206; TCFA, n = 205, calcified nodules, n = 147; cholesterol crystals, n = 225; plaque disruption, n = 266; erosion, n = 13; intraplaque hemorrhages, n = 203; and microchannels, n = 260). AMI was diagnosed in 13.5% of patients, and macrophages were detected in 79.9% of vessels. Interobserver reproducibility of macrophage arc measurements was excellent, with intraclass correlation coefficient (Cronbach’s alpha) of 0.956 (95% CI: 0.937-0.972) for mean arc, 0.942 (95% CI: 0.926-0.955) for maximum arc, and 0.938 (95% CI: 0.921-0.952) for MAS. Intraobserver reproducibility was similarly robust: intraclass correlation coefficients were 0.958 (95% CI: 0.945-0.968) for maximum arc, 0.963 (95% CI: 0.952-0.972) for mean arc, and 0.955 (95% CI: 0.942-0.965) for MAS. Semiquantitative grading of MAS (0-4 scale) demonstrated strong agreement, with interobserver κ = 0.89 (95% CI: 0.85-0.93) and intraobserver κ = 0.86 (95% CI: 0.79-0.91). The median lipid core arc was 204°, median maximum arc 133°, median mean arc 48.61°/mm, and median MAS 2.14. Clinical and procedural characteristics of the study population are summarized in Table 1.

**Table 1:** Patient and Lesion Characteristics (N = 1,129)

Age (y)	61 (53-69)
Female	344 (33.5)
LVEF (%)	63 (59-67)
Hypertension	638 (62.2)
Hypercholesterolemia	312 (30.4)
Smoking habit	635 (61.9)
Diabetes	339 (33.1)
Prior MI	159 (15.5)
Prior PCI	249 (24.3)
LDL-C (mmol/L)	2.1 (1.6-2.7)
LDL-C ≥2.6 mmol/L	294 (26.8)
OCT macrophage finding	Vessels (1,278)
Mean arc (°/mm)	51.39 (22.08-105.04)
Mean arc ≥78.89°/mm	275 (23.4)
MAS	1.68 (0.82-3.07)
MAS ≥2.34	378 (32.2)
Maximum arc (°)	134 (90-203)
Maximum arc ≥156.5°	402 (34.3)
Plaque characteristics	
TCFA	205 (17.5)
Plaque rupture	206 (17.6)
Maximum lipid core arc ≥186.5°	519 (44.2)
Plaque stratification	266 (22.7)
Intraplaque hemorrhage	203 (17.3)
Calcified nodules	147 (12.5)
Microchannel	260 (22.2)
Cholesterol crystals	225 (19.2)

LDL-C = low-density lipoprotein cholesterol; LVEF = left ventricular ejection fraction; MAS = mean arc score; MI = Myocardial Infarction; OCT = optical coherence tomography; PCI = percutaneous coronary intervention; TCFA = thin-cap fibroatheroma.

### Correlation between macrophage arc and vulnerable plaque

**Figure 2:**
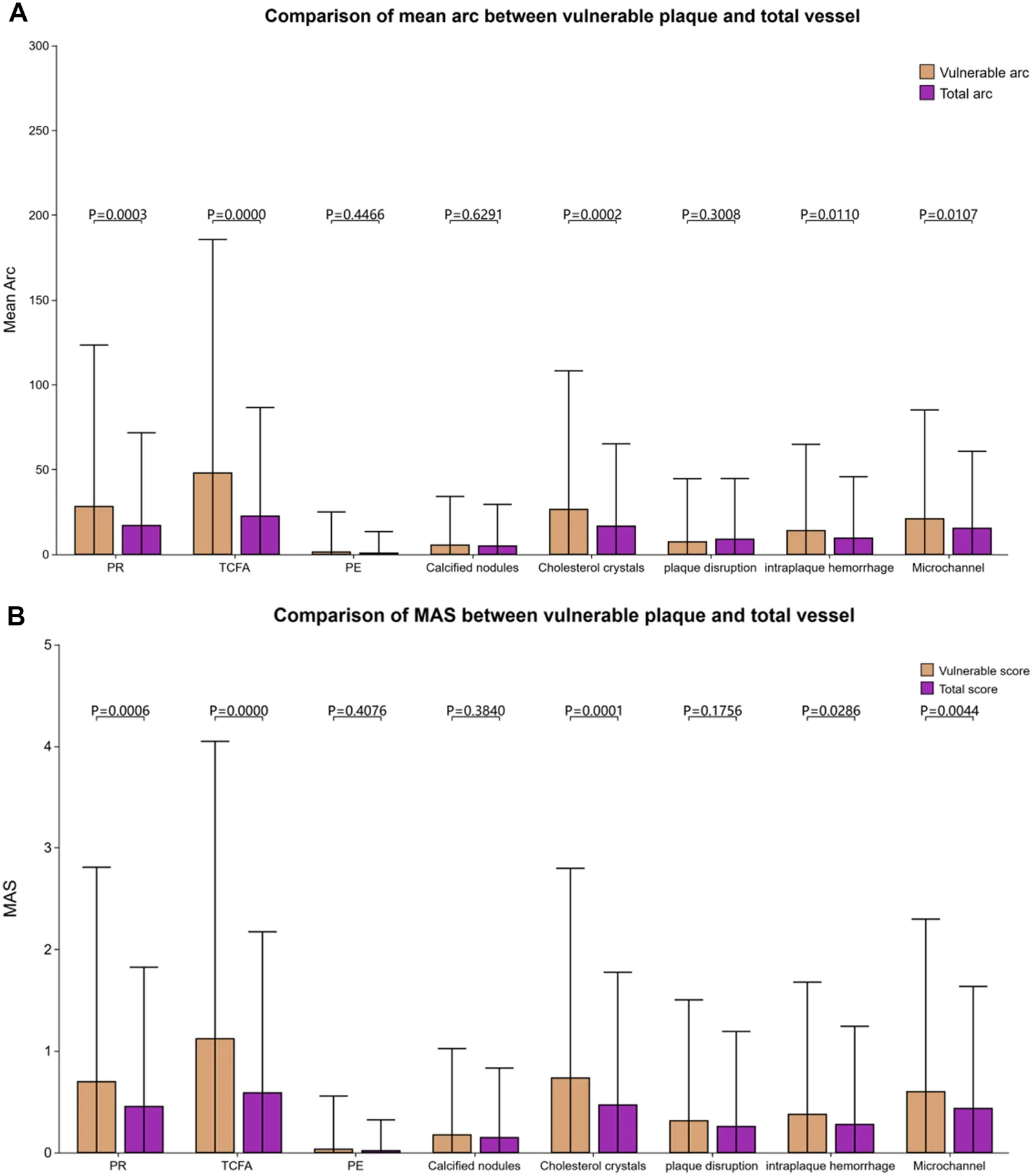
Comparison of Mean Macrophage Arc and Macrophage Activity Score (MAS) Between Regions Adjacent to Vulnerable Plaques and the Entire Vessel Segment (A) Mean macrophage arc; (B) Macrophage activity score (MAS). Both mean arc and MAS were significantly higher in regions near vulnerable plaques compared with the total vessel segment. Exact *P* values: PR-P<0.0001, TCFA *P* < 0.0001, cholesterol crystals *P* = 0.0001/0.0002, intraplaque hemorrhage *P* = 0.0110/0.0286, and microchannel *P* = 0.0107/0.0044. MAS = mean arc score; PE = plaque erosion; PR = plaque rupture; TCFA = thin-cap fibroatheroma.

The mean arc in regions adjacent to PR and TCFA (within 5 mm proximal and distal) was significantly greater than that observed across the entire vessel segment (*P* < 0.0001); similar patterns were observed for cholesterol crystallization, intraplaque hemorrhage, and microchannel (Figure 2A). Likewise, the MAS in regions near PR and TCFA was significantly higher than that observed across the entire vessel segment (*P* < 0.0001), with consistent findings for cholesterol crystals, intraplaque hemorrhage, and microchannels (Figure 2B).

Univariate and multivariate regression analyses identified several variables with associations to PR, TCFA, and AMI in this cohort. For PR, significant correlates comprised left ventricular ejection fraction, MAS, mean arc, and maximum arc, as presented in Table 2 and Supplemental Appendix.

**Table 2:** Logistic Regression Analysis for the Risk Factors of PR

	Univariate			Multivariate^a^		
	*P* Value	OR	95% CI	*P* Value	OR	95% CI
Age ≥65 y	0.84	0.97	0.70-1.33	0.82	1.39	0.717-1.487
Male	0.01	1.88	1.23-2.89	0.39	0.976	0.765-1.449
LVEF	<0.001	0.97	0.95-0.98	0.001	0.969	0.951-0.988
Hypertension	0.33	0.86	0.63-1.17	0.29	1.298	0.805-1.649
Hypercholesterolemia	0.33	1.18	0.85-1.65	0.92	1.021	0.675-1.492
Smoking habit	0.14	1.27	0.93-1.73	0.70	1.079	0.732-1.589
Diabetes	0.83	0.96	0.69-1.35	0.16	1.527	0.805-1.911
Prior MI	0.3	1.25	0.82-1.90	0.593	1.156	0.679-1.967
Prior PCI	0.49	0.86	0.56-1.33	0.696	0.909	0.561-1.471
LDL-C	0.34	0.82	0.55-1.23	0.542	1.538	0.762-1.954
OCT macrophage						
Mean arc	<0.0001	1.008	1.006-1.010	0.009	1.004	1.001-1.007
Mean arc ≥78.89°/mm	<0.0001	3.871	2.794-5.364	<0.001	2.724	1.600-4.636

PR = plaque rupture; other abbreviations as in Table 1.

a Each macrophage parameter (mean arc, maximum arc, MAS) was entered into separate multivariate models, not simultaneously into a single model; Hosmer-Lemeshow goodness-of-fit test value *P* = 0.229; Nagelkerke R-squared value R2 = 0.36; AUC value from ROC curves based on multivariate models C = 0.728; *P* values were adjusted using the Bonferroni method (α = 0.05/3 = 0.017).

TCFA was robustly associated with MAS, mean arc, and maximum arc (Table 3, Supplemental Appendix). Meanwhile, AMI was correlated with left ventricular ejection fraction, MAS, mean arc, maximum arc. (Table 4, Supplemental Appendix). Notably, mean arc, maximum arc, and MAS represented potential utility for PR, TCFA, and AMI, respectively.

**Table 3:** Logistic Regression Analysis for the Risk Factors of TCFA

	Univariate			Multivariate^a^		
	*P* Value	OR	95% CI	*P* Value	OR	95% CI
Age ≥65 y	0.61	0.92	0.67-1.27	0.981	0.995	0.654-1.514
Male	0.46	1.16	0.79-1.70	0.174	1.374	0.869-2.171
LVEF	0.005	0.97	0.95-0.98	0.028	0.976	0.956-0.997
Hypertension	0.95	0.99	0.72-1.35	0.854	0.986	0.848-1.147
Hypercholesterolemia	0.02	1.52	0.10-2.11	0.568	1.136	0.733-1.763
Smoking habit	0.89	0.98	0.72-1.33	0.999	1.000	0.655-1.528
Diabetes	0.33	1.18	0.85-1.63	0.571	0.883	0.574-1.358
Prior MI	0.64	1.11	0.72-1.71	0.890	1.042	0.581-1.870
Prior PCI	0.17	1.32	0.88-1.97	0.086	1.559	0.939-2.589
LDL-C	0.53	1.15	0.74-1.78	0.021	1.283	1.038-1.586
OCT macrophage						
Mean arc	<0.0001	1.016	1.013-1.019	<0.001	1.012	1.008-1.016
Mean arc ≥78.89°/mm	<0.0001	8.48	5.95-12.11	0.002	2.623	1.426-4.827

Abbreviations as in Table 1.

a Each macrophage parameter (mean arc, maximum arc, MAS) was entered into separate multivariate models, not simultaneously into a single model; Hosmer-Lemeshow goodness-of-fit test value *P* = 0.647; Nagelkerke R-squared value R2 = 0.46; AUC value from ROC curves based on multivariate models C = 0.841; *P* values were adjusted using the Bonferroni method (α = 0.05/4 = 0.0125).

**Table 4:** Logistic Regression Analysis for the Risk Factors of AMI

	Univariate			Multivariate^a^		
	*P* Value	OR	95% CI	*P* Value	OR	95% CI
Age ≥65 y	0.001	0.53	0.37-0.77	0.623	0.876	0.517-1.485
Male	<0.0001	2.35	1.44-3.83	0.086	1.659	0.930-2.960
LVEF	0.24	1.65	0.70-3.87	0.001	0.945	0.922-0.969
Hypertension	0.02	0.68	0.49-0.94	0.114	0.857	0.708-1.038
Hypercholesterolemia	0.08	1.36	0.96-1.93	0.327	1.304	0.767-2.216
Smoking habit	0.003	1.66	1.18-2.34	0.054	1.689	0.991-2.880
Diabetes	0.35	1.18	0.83-1.67	0.888	0.963	0.566-1.636
Prior MI	0.03	0.54	0.31-0.94	0.010	0.278	0.104-0.740
Prior PCI	0.0023	0.4	0.22-0.72	0.004	0.290	0.124-0.677
LDL-C	0.0012	2.54	1.40-4.58	0.677	1.060	0.806-1.393
Plaque characteristics						
The maximum lipid core arc ≥186.5°	<0.0001	0.44	0.23-0.85	0.812	0.934	0.529-1.647
Layered plaque	0.21	1.13	0.77-1.67	0.995	0.998	0.539-1.850
Intra-plaque hemorrhage	0.33	2.97	2.07-4.28	0.959	1.017	0.541-1.911
Calcified nodules	0.01	3.43	2.40-4.92	0.244	0.594	0.247-1.428
Microchannel	0.54	1.29	0.87-1.93	0.216	0.697	0.393-1.235
Cholesterol crystals	0.21	1.65	0.70-3.87	0.341	1.303	0.756-2.245
PR	<0.0001	0.53	0.37-0.77	0.183	1.468	0.835-2.582
TCFA	<0.0001	2.35	1.44-3.83	0.046	1.855	1.011-3.406
OCT macrophage						
Mean arc	<0.0001	1.009	1.006-1.011	0.006	1.001	1.001-1.007
Mean arc ≥78.89°/mm	<0.0001	2.25	1.62-4.01	<0.001	4.505	2.220-9.143

AMI = acute myocardial infarction; other abbreviations as in Tables 1 and 2.

a Each macrophage parameter (mean arc, maximum arc, MAS) was entered into separate multivariate models, not simultaneously into a single model; Hosmer-Lemeshow goodness-of-fit test value *P* = 0.274; Nagelkerke R-squared value R2 = 0.49; AUC value from ROC curves based on multivariate models C = 0.819; *P* values were adjusted using the Bonferroni method (α = 0.05/6 = 0.0083).

**Figure 3:**
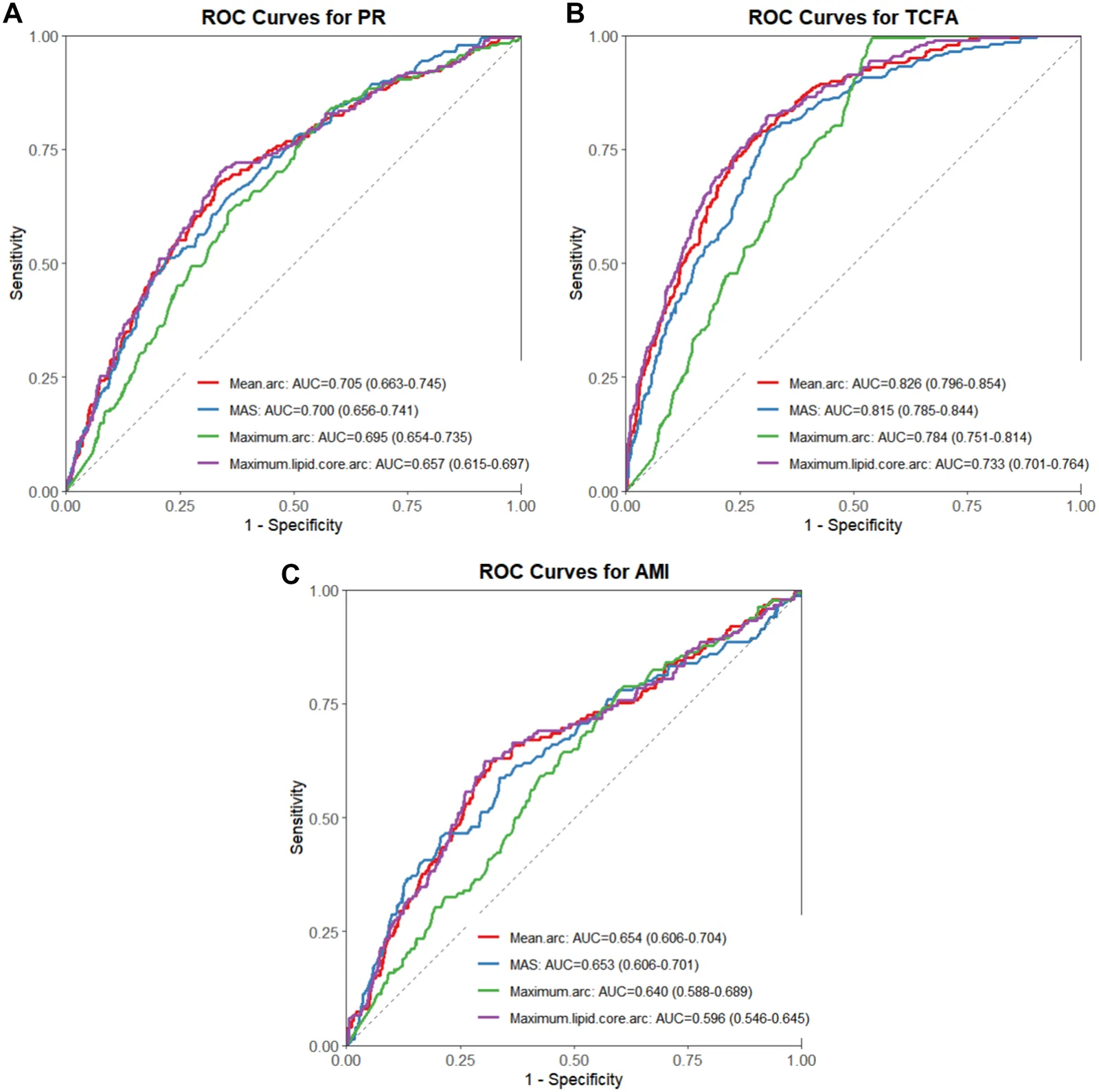
ROC Curve Analysis The ROC curves for the mean arc (red line), MAS (blue line), maximum arc (green line), and lipid core arc (purple line), showing correlative capacity to detect (A) PR, (B) TCFA, and (C) AMI. AMI = acute myocardial infarction; AUC = area under the curve; ROC = receiver operating characteristic; other abbreviations as in Figure 2.

Receiver operating characteristic curve analysis from the raw parameters was conducted to determine the optimal cutoff values for macrophage features in predicting PR, TCFA, and AMI. Optimal cutoff values were identified as follows: maximum arc ≥156.5°, mean arc ≥78.89°/mm, and MAS ≥2.34 (Figure 3).

**Central Illustration:**
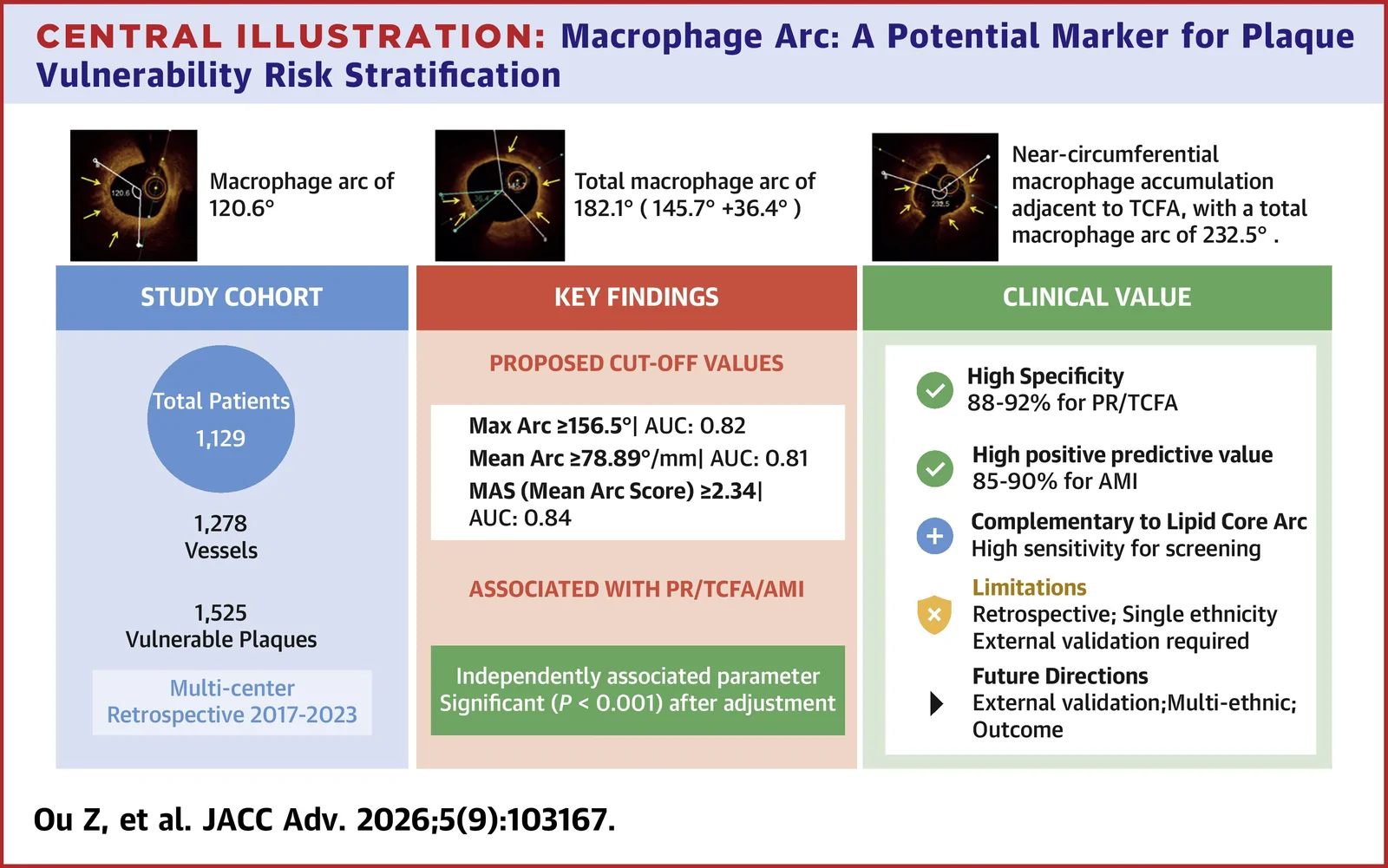
Macrophage Arc: A Potential Marker for Plaque Vulnerability Risk Stratification The central illustration outlines this multicenter retrospective analysis of OCT-derived macrophage arc parameters in 1129 CAD patients. The study defines candidate cutoffs (max arc ≥156.5°, mean arc ≥78.89°/mm, MAS ≥2.34) that are significantly associated with PR, TCFA, and AMI after multivariate adjustment. Macrophage arc parameters show high specificity and complement lipid core arc for improved two-step risk stratification in CAD. Critical limitations of retrospective nature and future directions for external validation are also indicated. AMI = acute myocardial infarction; MAS = mean arc score; PR = plaque rupture; TCFA= thin-cap fibroatheroma.

### Predictive value of macrophage arc vs maximum lipid core arc

The macrophage arc demonstrated favorable predictive performance compared with the lipid core arc in evaluating PR, TCFA, and AMI. Specifically, maximum arc, mean arc, and MAS showed better matrics than the lipid core arc in terms of area under the curve (AUC) value, specificity, and positive predictive value (PPV), although sensitivity was lower. Among the evaluated metrics, MAS ≥2.34 yielded the highest AUC, whereas a lipid core arc ≥186.5°provided the highest sensitivity and negative predictive value, and mean arc ≥78.89°/mm demonstrated the highest whole specificity and PPV (Table 5), DeLong’s test confirmed that macrophage arc parameters had significantly higher AUCs than lipid core arc (all *P* < 0.01) (Supplemental Appendix).

**Table 5:** Comparison Between the Lipid Core and Macrophage Arcs

	AUC (95% CI)	Sensitivity (95% CI)	Specificity (95% CI)	PPV	NPV
PR					
Lipid core arc ≥186.5°	0.66 (0.62-0.70)	0.79 (0.73-0.84)	0.47 (0.42-0.52)	0.28	0.81
Mean arc ≥78.89°/mm	0.71 (0.67-0.75)	0.70 (0.64-0.76)	0.66 (0.61-0.71)	0.33	0.89
Maximum arc ≥156.5°	0.70 (0.66-0.74)	0.64 (0.58-0.70)	0.65 (0.60-0.70)	0.3	0.88
MAS ≥2.34	0.70 (0.65-0.74)	0.67 (0.61-0.73)	0.67 (0.62-0.72)	0.32	0.89
TCFA					
Lipid core arc ≥186.5°	0.73 (0.69-0.77)	1.00 (0.98-1.00)	0.50 (0.45-0.55)	0.34	0.94
Mean arc ≥78.89°/mm	0.83 (0.79-0.87)	0.81 (0.75-0.86)	0.69 (0.64-0.74)	0.42	0.92
Maximum arc ≥156.5°	0.78 (0.74-0.82)	0.79 (0.73-0.84)	0.69 (0.64-0.74)	0.38	0.92
MAS ≥2.34	0.82 (0.78-0.86)	0.79 (0.73-0.84)	0.70 (0.65-0.75)	0.41	0.92
AMI					
Lipid core arc ≥186.5°	0.60 (0.55-0.65)	0.74 (0.67-0.80)	0.44 (0.39-0.49)	0.19	0.9
Mean arc ≥78.89°/mm	0.65 (0.60-0.70)	0.60 (0.53-0.67)	0.70 (0.65-0.75)	0.26	0.91
Maximum arc ≥156.5°	0.64 (0.59-0.69)	0.60 (0.53-0.67)	0.64 (0.59-0.69)	0.23	0.9
MAS ≥2.34	0.65 (0.60-0.70)	0.60 (0.53-0.67)	0.68 (0.63-0.73)	0.25	0.91

AUC = area under the curve; NPV = negative predictive value; PPV = positive predictive value; other abbreviations as in Tables 1, 2 and 4.

## Discussion

This multicenter, large-scale retrospective study suggests an association between macrophage arc parameters (maximum arc, mean arc, and MAS) and coronary plaque vulnerability, as well as AMI on admission. Our findings indicate that macrophage arc values (maximum arc ≥156.5°, mean arc ≥78.89°/mm, and MAS ≥2.34) serve as associated factors of PR, TCFA, and AMI. Moreover, these parameters showed favorable predictive performance relative to lipid core arc, with higher AUC, specificity, and PPV, although sensitivity was lower (Central Illustration). Thus, the comparative advantage of macrophage arc remains preliminary and should be interpreted with caution.

### Macrophage maximum arc: focal inflammation as a marker of plaque instability

Local macrophage infiltration is a well-recognized driver of plaque destabilization, with proinflammatory macrophages promoting matrix degradation and fibrous cap thinning through metalloproteinase secretion.^22^^,^^23^ The maximum macrophage arc, reflecting focal inflammatory burden at the most macrophage-dense cross section, has previously been associated with non–ST-segment elevation acute coronary syndromes^20^; however, its correlation with specific vulnerable plaque features (such as PR and TCFA) has not yet been established. Our study addresses this gap by demonstrating that a maximum arc ≥156.5° is correlated with PR, TCFA, AMI, and secondary vulnerable features (including cholesterol crystals and microchannels). These findings are consistent with the CLIMA study subanalysis,^14^ which validated the reproducibility of OCT-based macrophage arc quantification and its association with adverse clinical outcomes. As a rapidly measurable OCT parameter, maximum arc provides real-time insight into focal arterial wall inflammation, making it a practical tool for the intraprocedural assessment of plaque instability during percutaneous coronary intervention.

Notably, the retrospective design of our study limits the ability to establish a causal relationship between focal macrophage accumulation and PR; however, the consistency of our findings with preclinical studies^5^^,^^22^ and smaller human cohorts^20^ reinforces the clinical relevance of maximum arc as a marker of focal inflammatory activity.

### Macrophage mean arc and MAS: improvement in evaluating diffuse inflammation

A key advance of this study is the introduction of mean arc (total macrophage arc per mm of vessel segment) and MAS (cumulative macrophage grade per mm of vessel segment), which address critical limitations of the previously used average arc (total macrophage arc divided by the number of OCT frames).^14^^,^^15^^,^^24^, ^25^, ^26^ The principle advantage of mean arc/MAS is their vessel length normalization, a feature absent in average arc quantification. Because OCT pull backs capture coronary segments of varying lengths (typically 10-50 mm in clinical practice), and average arc—by dividing by frame count rather than physical length—fails to account for differences in pull back speed or frame density, resulting in inconsistent quantification of macrophage infiltration across different vessel segments. In contrast, mean arc and MAS standardize macrophage burden to millimeters of imaged vessel, enabling direct comparison of inflammatory levels across patients, vessels, and clinical centers. This normalization is clinically important for evaluating the “vulnerable vessel” concept, as acute coronary syndromes are increasingly recognized to involve multifocal inflammation rather than isolated plaque pathology.^27^, ^28^, ^29^, ^30^ Our finding that mean arc and MAS are significantly higher in PR/TCFA-adjacent regions compared with the entire vessel segment (*P* < 0.0001) validates their ability to detect localized inflammatory hotspots within a broader vascular bed—an outcome that cannot be captured by average arc due to its lack of spatial normalization.

### Clinical implications of high-specificity macrophage arc

A defining characteristic of macrophage arc parameters (mean arc ≥78.89°/mm, maximum arc ≥156.5°, and MAS ≥2.34) is their high specificity and low sensitivity for predicting PR, TCFA, and AMI—demonstrating an inverse pattern compared with the lipid core arc. The high sensitivity of lipid core arc makes it an ideal tool for ruling out vulnerable plaques, as a negative result effectively excludes the presence of a large lipid core and low plaque stability.^31^ Specifically, macrophage arc parameters demonstrated significantly higher specificity, PPV, and AUC than lipid core arc, making them more suitable for “ruling in” high-risk patients in clinical practice. Conversely, lipid core arc showed higher sensitivity and negative predictive value, which is more appropriate for initial risk screening in the general population. Thus, the 2 markers provide complementary value in different clinical scenarios, rather than representing an absolute “superior or inferior” relationship.

### Subjective nature of macrophage identification limit application of cutoff values

A major challenge to the clinical translation of precise macrophage arc cutoff values is the subjective nature of OCT-based macrophage identification—a limitation inherent to all current OCT-based quantification methods.^10^^,^^11^ Signal interference from other plaque components and the absence of standardized grading criteria introduce variability, such that the same OCT image may yield different MAS/arc measurements across centers. Future prospective studies incorporating centralized image core laboratories and standardized macrophage identification protocols^17^ will be essential to refine these cutoff values and enhance their reproducibility.

### Study Limitations

The present study has several limitations. First, the present study has a retrospective design, which inherently brings certain limitations to the methodology chosen. Second, existing literature lacks robust methodologies for accurately distinguishing M1 from M2 macrophages using OCT, marking a critical area for further investigation. Third, in our study, only the target vessel and segment were evaluated by OCT. Fourth, this study detected macrophage, not in a direct histopathological way. Finally, the proposed cutoff values are candidate predictive markers, which require further validation in independent external cohorts before clinical application.

## Conclusion

Macrophage arcs exhibit an association with plaque vulnerability and AMI. Although candidate cutoffs (maximum arc ≥156.5°, mean arc ≥78.89°/mm, and MAS ≥2.34) offer potential specificity/PPV for plaque vulnerability and AMI, further validation in independent external cohorts is necessary.

Perspectives **COMPETENCY IN MEDICAL KNOWLEDGE:** This work helps clinicians expand their understanding of OCT-detected plaque inflammation. We developed length-normalized macrophage arc indicators and confirmed their diagnostic thresholds for TCFA, plaque rupture and acute myocardial infarction. These tools address key drawbacks of classic NSD quantification and create a combined risk evaluation strategy that integrates inflammatory and lipid plaque features.

**TRANSLATIONAL OUTLOOK:** Reliance on manual visual assessment of macrophages leads to variable readings between medical centers. Follow-up research should build AI-based automatic OCT analysis tools and verify our cutoffs in independent prospective cohorts. Macrophage arc measurements may also act as a substitute outcome marker for anti-inflammatory atherosclerosis trials, supporting more targeted intracoronary imaging practice.

## Funding support and author disclosures

This work was supported by the Chongqing Health Appropriate Technology Promotion Project (2024jstg) and Scientific and Technological Innovation Project of the Army Medical University (2022XJS26). The authors have reported that they have no relationships relevant to the contents of this paper to disclose.
