# Supplementary material for: Relationship Between Macrophage Arc and Plaque Vulnerability in Patients With Coronary Artery Disease
Source: JACC Adv. 2026 Aug 19;5(9):103167. doi: 10.1016/j.jacadv.2026.103167 (PMC13521088; doi:10.1016/j.jacadv.2026.103167)
Supplement: Supplementary file 1 [file mmc1.docx]

Supplementary

SOP

Complete separation of lesion delineation and macrophage measurement in both workflow and personnel

Step 1 (Lesion Annotation): Two independent investigators who did not participate in any macrophage arc measurement completed the full-vessel OCT annotation. They predefined and locked all the target regions, including the longitudinal range of each target plaque, the core frame of PR/TCFA, the 5-mm adjacent segment boundary, and the exclusion frames with side branches or artifacts, via consensus reading and formed a fixed, frame-locked measurement template. This step did not involve macrophage assessment or measurement.

Step 2 (Macrophage Measurement): The two investigators responsible for macrophage arc measurement only received the frame-locked target region template and were strictly blinded to all patient clinical information (including AMI diagnosis) and the final diagnostic results of the plaque phenotype (whether the target region was PR/TCFA or other lesion types). They only needed to complete the macrophage identification and arc measurement in the predefined fixed frames according to the standardized SOP without any knowledge of the vulnerability grade of the measured plaque. This design effectively eliminates subjective bias arising from investigators' prior knowledge of the plaque’s high-risk phenotype.

Extract all valid frames from the target plaque segment in the longitudinal order (from the proximal to distal end of the plaque). Do not skip any valid frames—scanning must be continuous and comprehensive.

Label frames sequentially (e.g., Frame 1, Frame 2, ..., Frame N) for traceability.

2.2 Individual Frame Macrophage Arc Measurement

For each valid frame (repeat Steps 2.2.1–2.2.3 for all frames):

2.2.1 Macrophage Signal Identification

Visually inspect the frame for macrophage-specific signals, defined as follows:

Signal-rich, discrete, or confluent punctate regions

Intensity ≥ 30% above background speckle noise

No distinct anatomical boundaries (distinguished from calcification [high intensity + shadowing], cholesterol crystals [thin linear signals], or microchannels [signal-poor cavities]).

Mark all valid macrophage signal regions in the frame (use software annotation tools if available).

2.2.2 Circumferential Arc Calculation

Use the workstation’s angle measurement tool, with the vessel lumen center as the fixed origin:

Identify the proximal start point (clockwise direction) of the macrophage signal region

Identify the distal end point (clockwise direction) of the macrophage signal region

Draw a circumferential arc connecting the start and end points and record the angle value (in degrees, e.g., 125°)

If multiple nonconsecutive macrophage clusters exist in one frame, calculate the arc angle for each cluster separately, sum the angles, and record the total as the frame’s macrophage arc value.

2.2.3 Log the Value

Enter the frame number and corresponding macrophage arc value into the measurement log.

If a frame has no detectable macrophages, record the arc value as 0°.

2.3 Extract Maximum Arc Value

After completing frame-by-frame measurement for all valid frames in the target segment, review the measurement log.

Identify the highest arc value from the frame-by-frame dataset—this is the “maximum macrophage arc” for the target plaque segment.

Record the maximum value and the corresponding frame number (for reproducibility verification) in the log.

**Collinearity analysis**

**Collinearity analysis for mean arc and features**

|  | **Collinearity analysis** | |
| --- | --- | --- |
|  | **Tolerance** | **VIF** |
| Age ≥ 65 | 0.900 | 1.111 |
| Male | 0.852 | 1.174 |
| LVEF | 0.914 | 1.094 |
| Hypertension | 0.935 | 1.070 |
| Hypercholesterolemia | 0.893 | 1.120 |
| Smoking habit | 0.844 | 1.185 |
| Diabetes | 0.937 | 1.067 |
| Prior MI | 0.812 | 1.231 |
| Prior PCI | 0.797 | 1.255 |
| LDL-C | 0.811 | 1.233 |
| Plaque characteristics |  |  |
| The maximum lipid core arc ≥ 186.5° | 0.842 | 1.188 |
| Layered plaque | 0.956 | 1.046 |
| Intraplaque  haemorrhage | 0.945 | 1.058 |
| Calcified nodules | 0.952 | 1.051 |
| Microchannel | 0.945 | 1.058 |
| Cholesterol crystals | 0.925 | 1.082 |
| PR | 0.799 | 1.251 |
| TCFA | 0.645 | 1.551 |
| OCT macrophage |  |  |
| Mean arc | 0.379 | 2.637 |
| Mean arc ≥ 78.89°/mm | 0.393 | 2.543 |

**Collinearity analysis for maximum arc and features**

|  | **Collinearity analysis** | |
| --- | --- | --- |
|  | **Tolerance** | **VIF** |
| Age ≥ 65 | 0.901 | 1.110 |
| Male | 0.863 | 1.159 |
| LVEF | 0.916 | 1.092 |
| Hypertension | 0.935 | 1.070 |
| Hypercholesterolemia | 0.893 | 1.120 |
| Smoking habit | 0.841 | 1.190 |
| Diabetes | 0.940 | 1.063 |
| Prior MI | 0.810 | 1.235 |
| Prior PCI | 0.802 | 1.246 |
| LDL-C | 0.818 | 1.222 |
| Plaque characteristics |  |  |
| The maximum lipid core arc ≥ 186.5° | 0.814 | 1.228 |
| Layered plaque | 0.959 | 1.043 |
| Intraplaque  haemorrhage | 0.944 | 1.059 |
| Calcified nodules | 0.952 | 1.050 |
| Microchannel | 0.942 | 1.061 |
| Cholesterol crystals | 0.927 | 1.079 |
| PR | 0.798 | 1.254 |
| TCFA | 0.691 | 1.446 |
| OCT macrophage |  |  |
| Maximum arc | 0.294 | 3.406 |
| Maximum arc ≥ 156.5 | 0.312 | 3.207 |
|  |  |  |

**Collinearity analysis for MAS and features**

|  | **Collinearity analysis** | |
| --- | --- | --- |
|  | **Tolerance** | **VIF** |
| Age ≥ 65 | 0.901 | 1.109 |
| Male | 0.848 | 1.179 |
| LVEF | 0.916 | 1.092 |
| Hypertension | 0.933 | 1.072 |
| Hypercholesterolemia | 0.892 | 1.121 |
| Smoking habit | 0.844 | 1.185 |
| Diabetes | 0.936 | 1.069 |
| Prior MI | 0.812 | 1.231 |
| Prior PCI | 0.794 | 1.259 |
| LDL-C | 0.819 | 1.222 |
| Plaque characteristics |  |  |
| The maximum lipid core arc ≥ 186.5° | 0.850 | 1.177 |
| Layered plaque | 0.956 | 1.046 |
| Intraplaque  haemorrhage | 0.944 | 1.059 |
| Calcified nodules | 0.952 | 1.050 |
| Microchannel | 0.942 | 1.062 |
| Cholesterol crystals | 0.920 | 1.087 |
| PR | 0.801 | 1.249 |
| TCFA | 0.652 | 1.534 |
| OCT macrophage |  |  |
| MAS | 0.339 | 2.951 |
| MAS ≥ 2.34 | 0.351 | 2.852 |
|  |  |  |

**Multivariate logistic regression analysis**

**Supplementary Table. Multivariate logistic regression analysis for the risk factors of PR**

|  | ***P* value** | **OR** | **95%CI** |
| --- | --- | --- | --- |
|  |  |  |  |
| Age ≥ 65 | 0.875 | 1.031 | 0.704-1.509 |
| Male | 0.331 | 0.812 | 0.533–1.236 |
| LVEF | 0.005 | 0.973 | 0.955-0.992 |
| Hypertension | 0.251 | 0.921 | 0.801–1.06 |
| Hypercholesterolemia | 0.891 | 1.029 | 0.687–1.541 |
| Smoking habit | 0.480 | 1.15 | 0.78–1.696 |
| Diabetes | 0.305 | 0.814 | 0.549–1.207 |
| Prior MI | 0.668 | 1.123 | 0.66–1.912 |
| Prior PCI | 0.435 | 0.826 | 0.511–1.335 |
| LDL-C | 0.784 | 0.971 | 0.788–1.197 |
| OCT macrophage |  |  |  |
| Maximum arc | <0.001 | 1.008 | 1.004-1.012 |
| Maximum arc ≥ 156.5 | 0.004 | 2.257 | 1.279-5.142 |

Hosmer–Lemeshow goodness-of-fit test value P = 0.501; Nagelkerke R-squared value R2 = 0.42; AUC value from ROC curves based on multivariate models C = 0.702

|  | ***P* value** | **OR** | **95%CI** |
| --- | --- | --- | --- |
|  |  |  |  |
| Age ≥ 65 | 0.86 | 1.53 | 0.708-1.512 |
| Male | 0.962 | 1.010 | 0.661-1.543 |
| LVEF | 0.003 | 0.972 | 0.954-0.990 |
| Hypertension | 0.252 | 1.129 | 0.800-1.469 |
| Hypercholesterolemia | 0.754 | 1.066 | 0.713-1.594 |
| Smoking habit | 0.701 | 1.078 | 0.733-1.585 |
| Diabetes | 0.154 | 1.479 | 0.503-1.987 |
| Prior MI | 0.569 | 1.168 | 0.685-1.993 |
| Prior PCI | 0.723 | 0.916 | 0.565-1.486 |
| LDL-C | 0.703 | 1.169 | 0.784-1.679 |
| OCT macrophage |  |  |  |
| MAS | 0.01 | 1.205 | 1.046-1.388 |
| MAS ≥ 2.34 | 0.002 | 2.404 | 1.368-4.225 |

Hosmer–Lemeshow goodness-of-fit test value P = 0.305; Nagelkerke R-squared value R2 = 0.52; AUC value from ROC curves based on multivariate models C = 0.700

**Supplementary Table. Multivariate logistic regression analysis for the risk factors of TCFA**

|  | **P value** | **OR** | **95%CI** |
| --- | --- | --- | --- |
|  |  |  |  |
| Age ≥ 65 | 0.747 | 1.069 | 0.715-1.598 |
| Male | 0.988 | 1.003 | 0.653-1.541 |
| LVEF | 0.058 | 0.980 | 0.960-1.001 |
| Hypertension | 0.677 | 0.970 | 0.839-1.121 |
| Hypercholesterolemia | 0.696 | 1.087 | 0.714-1.655 |
| Smoking habit | 0.486 | 1.156 | 0.769-1.738 |
| Diabetes | 0.922 | 0.980 | 0.652-1.472 |
| Prior MI | 0.996 | 0.999 | 0.567-1.760 |
| Prior PCI | 0.269 | 1.316 | 0.809-2.140 |
| LDL-C | 0.005 | 1.349 | 1.097-1.659 |
| OCT macrophage |  |  |  |
| Maximum arc | 0.001 | 1.009 | 1.005-1.012 |
| Maximum arc ≥ 156.5 | 0.003 | 2.669 | 1.401-5.085 |

Hosmer–Lemeshow goodness-of-fit test value P = 0.289; Nagelkerke R-squared value R2 = 0.39; AUC value from ROC curves based on multivariate models C = 0.822

|  | **P value** | **OR** | **95%CI** |
| --- | --- | --- | --- |
| Age ≥ 65 | 0.773 | 1.063 | 0.702-1.610 |
| Male | 0.056 | 1.555 | 0.988-2.447 |
| LVEF | 0.032 | 0.977 | 0.957-.998 |
| Hypertension | 0.726 | 0.974 | 0.8381.131- |
| Hypercholesterolemia | 0.402 | 1.203 | 0.781-1.852 |
| Smoking habit | 0.999 | 1.000 | 0.659-1.517 |
| Diabetes | 0.377 | 0.825 | 0.538-1.264 |
| Prior MI | 0.892 | 1.041 | 0.580-1.870 |
| Prior PCI | 0.060 | 1.630 | 0.980-2.712 |
| LDL-C | 0.013 | 1.303 | 1.058-1.604 |
| OCT macrophage |  |  |  |
| MAS | <0.001 | 1.611 | 1.367-1.900 |
| MAS ≥ 2.34 | 0.002 | 2.626 | 1.414-4.875 |

Hosmer–Lemeshow goodness-of-fit test value P = 0.676; Nagelkerke R-squared value R2 = 0.34; AUC value from ROC curves based on multivariate models C = 0.809

**Supplementary Table. Multivariate logistic regression analysis for the risk factors of AMI**

|  | ***P* value** | **OR** | **95%CI** |
| --- | --- | --- | --- |
| Age ≥ 65 | 0.739 | 0.915 | 0.542-1.545 |
| Male | 0.242 | 1.395 | 0.799-2.434 |
| LVEF | 0.001 | .952 | 0.929-0.975 |
| Hypertension | 0.101 | .853 | 0.706-1.032 |
| Hypercholesterolemia | 0.327 | 1.298 | 0.770-2.189 |
| Smoking habit | 0.054 | 1.682 | 0.991-2.855 |
| Diabetes | 0.809 | 1.066 | 0.634-1.791 |
| Prior MI | 0.016 | 0.303 | 0.114-0.803 |
| Prior PCI | 0.004 | 0.299 | 0.130-0.685 |
| LDL-C | 0.485 | 1.103 | 0.837-1.454 |
| Plaque characteristics |  |  |  |
| The maximum lipid core arc ≥ 186.5° | 0.784 | 0.924 | 0.525-1.627 |
| Layered plaque | 0.935 | 0.975 | 0.536-1.777 |
| Intraplaque  haemorrhage | 0.952 | 0.981 | 0.528-1.822 |
| Calcified nodules | 0.270 | 0.616 | 0.261-1.457 |
| Microchannel | 0.346 | 0.765 | 0.438-1.336 |
| Cholesterol crystals | 0.324 | 1.309 | 0.767-2.236 |
| PR | 0.093 | 1.608 | 0.924-2.799 |
| TCFA | 0.007 | 2.233 | 1.241-4.017 |
| OCT macrophage |  |  |  |
| Maximum arc | .009 | 1.004 | 1.001-1.008 |
| Maximum arc ≥ 156.5 | 0.028 | 1.855 | 1.071-3.213 |

Hosmer–Lemeshow goodness-of-fit test value P = 0.244; Nagelkerke R-squared value R2 = 0.55; AUC value from ROC curves based on multivariate models C = 0.793

|  | ***P* value** | **OR** | **95%CI** |
| --- | --- | --- | --- |
| Age ≥ 65 | 0.688 | 0.898 | 0.530-1.521 |
| Male | 0.174 | 1.477 | 0.841-2.591 |
| LVEF | 0.000 | 0.950 | 0.927-.973 |
| Hypertension | 0.128 | 0.862 | 0.713-1.043 |
| Hypercholesterolemia | 0.305 | 1.314 | 0.780-2.214 |
| Smoking habit | 0.070 | 1.627 | 0.960-2.757 |
| Diabetes | 0.950 | 1.017 | 0.603-1.716 |
| Prior MI | 0.025 | 0.334 | 0.128-.874 |
| Prior PCI | 0.007 | .315 | 0.136-.729 |
| LDL-C | 0.394 | 1.126 | 0.857-1.481 |
| Plaque characteristics |  |  |  |
| The maximum lipid core arc ≥ 186.5° | 0.962 | 1.014 | 0.580-1.771 |
| Layered plaque | 0.899 | 1.040 | 0.572-1.891 |
| Intraplaque  haemorrhage | 0.940 | 0.976 | 0.524-1.820 |
| Calcified nodules | 0.339 | 0.657 | 0.277-1.557 |
| Microchannel | 0.356 | 0.769 | 0.440-1.343 |
| Cholesterol crystals | 0.348 | 1.293 | 0.756-2.212 |
| PR | 0.085 | 1.630 | 0.935-2.843 |
| TCFA | 0.022 | 2.018 | 1.105-3.686 |
| OCT macrophage | 0.002 | 1.230 | 1.082-1.398 |
| MAS | 0.002 | 1.230 | 1.082-1.398 |
| MAS ≥ 2.34 | <0.001 | 3.749 | 2.137-6.579 |

Hosmer–Lemeshow goodness-of-fit test value P = 0.557; Nagelkerke R-squared value R2 = 0.48; AUC value from ROC curves based on multivariate models C = 0.800

Deong’s Test

|  | 95%CI | Z value | P value |
| --- | --- | --- | --- |
| Mean arc vs. Lipid core arc | 0.04-0.163 | 3.252 | 0.0011 |
| MAS vs. Lipid core arc | 0.036-0.161 | 3.069 | 0.0021 |
| Maximum arc vs. Lipid core arc | 0.024-0.139 | 2.798 | 0.0051 |

STROBE Statement—Checklist of items that should be included in reports of ***cross-sectional studies***

|  | Item No | Recommendation |
| --- | --- | --- |
| **Title and abstract** | 1 | (*a*) Multicenter, retrospective cross‑sectional registry study. |
|  |  | (*b*) This multicenter OCT study of 1,129 CAD patients shows macrophage arc parameters (max arc ≥156.5°, mean arc ≥78.89°, MAS ≥2.34) are associated with plaque rupture, TCFA, and AMI. Macrophage arc complements lipid core arc for risk stratification |
| Introduction | | |
| Background/rationale | 2 | Macrophages are key inflammatory mediators detectable by OCT, but its association with plaque vulnerability and AMI remains unclear. |
| Objectives | 3 | To evaluate the association between OCT‑derived macrophage arc parameters and vulnerable plaque features and AMI |
| Methods | | |
| Study design | 4 | Multicenter, retrospective, cross‑sectional registry study. |
| Setting | 5 | Two tertiary centers (Xinqiao Hospital, Southwest Hospital, Army Medical University, Chongqing, China); Jan 2017–Apr 2023. |
| Participants | 6 | Eligibility: Consecutive CAD patients undergoing coronary angiography + OCT.  Exclusion: Poor OCT quality; effective vessel length <20 mm.  Enrolled: 1,129 patients / 1,278 vessels / 1,525 vulnerable plaques. |
| Variables | 7 | Outcomes: PR, TCFA (vessel‑level); AMI (patient‑level).  Exposures: Max arc (°), mean arc (°/mm), MAS, lipid core arc (°).  Confounders: Age, sex, LVEF, hypertension, diabetes, hypercholesterolemia, smoking, prior MI/PCI. |
| Data sources/ measurement | 8* | OCT: ILUMIEN/OPTIS systems; full‑vessel analysis.  Macrophage arc: Frame‑by‑frame; max arc, mean arc (total arc / vessel length),  MAS (0–4 grade sum / length).  Plaque features: PR, TCFA, lipid core arc per consensus definitions. |
| Bias | 9 | Blinded OCT analysis; independent readers + adjudication.  Standardized SOP; centralized measurement protocol. |
| Study size | 10 | Consecutive enrollment over 6 years; no formal power calculation; sample reflects real‑world OCT registry. |
| Quantitative variables | 11 | Continuous: Max arc, mean arc, MAS, lipid core arc, LVEF, LDL‑C. |
| Statistical methods | 12 | (*a*) t‑test / Mann‑Whitney U / χ²; Bonferroni correction. |
|  |  | (*b*) Logistic regression; separate models for each macrophage parameter (no multicollinearity); predefined + univariate covariates; DeLong’s test |
|  |  | (*c*) the overall missingness rate was <3.2%, determined that the missing data type was completely missing at random, and deleted this part of the data |
|  |  |  |
|  |  |  |
| Results | | |
| Participants | 13* | Screened: 1,307 patients / 1,436 vessels.  Included: 1,129 patients / 1,278 vessels. |
|  |  | Excluded: Poor quality / short vessel. |
|  |  |  |
| Descriptive data | 14* | (a) Table 1:Patient and lesion characteristics |
|  |  | (b) missing data: 62 |
| Outcome data | 15* | PR: n=206 (17.6%); TCFA: n=205 (17.5%); AMI: n=152(13.5%). |
| Main results | 16 | (*a*) Table 2-4. |
|  |  | (*b*) Cutoffs: Max ≥156.5°, mean ≥78.89°/mm, MAS ≥2.34. |
|  |  |  |
| Other analyses | 17 | Inter/intra‑observer reproducibility; DeLong test for AUC comparison; Sensitivity: Separate models per macrophage parameter. |
| Discussion | | |
| Key results | 18 | OCT‑derived macrophage arc parameters are independently associated with PR, TCFA, and AMI |
| Limitations | 19 | Retrospective, single‑country; OCT macrophage identification is subjective; No histology validation; Cutoffs need external validation. |
| Interpretation | 20 | Macrophage arc reflects coronary inflammation and complements lipid core arc for two‑step risk stratification. |
| Generalisability | 21 | Multicenter real‑world OCT cohort; findings need external prospective validation. |
| Other information | | |
| Funding | 22 | Chongqing Health Appropriate Technology Promotion Project (2024jstg); Army Medical University Innovation Project (2022XJS26). |

*Give information separately for exposed and unexposed groups.

**Note:** An Explanation and Elaboration article discusses each checklist item and gives methodological background and published examples of transparent reporting. The STROBE checklist is best used in conjunction with this article (freely available on the Web sites of PLoS Medicine at http://www.plosmedicine.org/, Annals of Internal Medicine at http://www.annals.org/, and Epidemiology at http://www.epidem.com/). Information on the STROBE Initiative is available at www.strobe-statement.org.
